# Acute hematologic toxicity of radiation therapy – a comprehensive analysis and predictive nomogram

**DOI:** 10.1093/jrr/rrad069

**Published:** 2023-09-22

**Authors:** Kazuya Takeda, Rei Umezawa, Takaya Yamamoto, Noriyoshi Takahashi, Yu Suzuki, Keita Kishida, So Omata, Keiichi Jingu

**Affiliations:** Department of Radiation Oncology, Tohoku University Graduate School of Medicine, 1-1 Seiryo-machi, Aoba-ku, Sendai, Miyagi 980-8574, Japan; Department of Radiation Oncology, South Miyagi Medical Center, 38-1 Nishi, Ogawara, Shibata, Miyagi 989-1253, Japan; Department of Radiation Oncology, Tohoku University Graduate School of Medicine, 1-1 Seiryo-machi, Aoba-ku, Sendai, Miyagi 980-8574, Japan; Department of Radiation Oncology, Tohoku University Graduate School of Medicine, 1-1 Seiryo-machi, Aoba-ku, Sendai, Miyagi 980-8574, Japan; Department of Radiation Oncology, Tohoku University Graduate School of Medicine, 1-1 Seiryo-machi, Aoba-ku, Sendai, Miyagi 980-8574, Japan; Department of Radiation Oncology, Tohoku University Graduate School of Medicine, 1-1 Seiryo-machi, Aoba-ku, Sendai, Miyagi 980-8574, Japan; Department of Radiation Oncology, Tohoku University Graduate School of Medicine, 1-1 Seiryo-machi, Aoba-ku, Sendai, Miyagi 980-8574, Japan; Department of Radiation Oncology, Tohoku University Graduate School of Medicine, 1-1 Seiryo-machi, Aoba-ku, Sendai, Miyagi 980-8574, Japan; Department of Radiation Oncology, Tohoku University Graduate School of Medicine, 1-1 Seiryo-machi, Aoba-ku, Sendai, Miyagi 980-8574, Japan

**Keywords:** radiotherapy, leukocytopenia, lymphocytopenia, anemia, thrombocytopenia

## Abstract

To investigate radiation-induced cytopenia and establish predictive nomograms for hematological toxicity, we reviewed 3786 patients aged 18 or older who received radiation monotherapy between 2010 and 2021 for non-hematologic malignancies. We collected data on patient background, treatment content and hematologic toxicities for 12 weeks after the start of radiotherapy. The patients were randomly divided into training and test groups in 7:3 ratio. In the training group, we conducted ordered logistic regression analysis to identify predictive factors for neutropenia, lymphocytopenia, anemia and thrombocytopenia. Nomograms to predict Grade 2–4 cytopenia were generated and validated in the test group. Grade 3 or higher hematologic toxicities were observed in 9.7, 44.6, 8.3 and 3.1% of patients with neutropenia, lymphocytopenia, anemia and thrombocytopenia, respectively. We identified six factors for neutropenia grade, nine for lymphocytopenia grade and six for anemia grade with statistical significance. In the analysis of thrombocytopenia, the statistical model did not converge because of a small number of events. Nomograms were generated using factors with high predictive power. In evaluating the nomograms, we found high area under the receiver operating characteristic curve values (neutropenia; 0.75–0.85, lymphopenia; 0.89–0.91 and anemia; 0.85–0.86) in predicting Grade 2–4 cytopenia in the test group. We established predictive nomograms for neutropenia, leukocytopenia and anemia and demonstrated high reproducibility when validated in an independent cohort of patients.

## INTRODUCTION

Hematologic toxicity is a well-known side effect of radiation therapy. Bone marrow irradiation can damage hematopoietic stem cells, and radiation-induced inflammation can suppress hematopoiesis [1]. Among the differentiated blood cells, which are generally radioresistant, lymphocytes have high radiosensitivity and undergo interphase death when passing through a radiation treatment field. Hematologic toxicities can interrupt radiotherapy and limit the use of concurrent and subsequent chemotherapy, which can affect patient outcomes [2–6].

Therefore, uncovering the predictive factors of radiation-induced hematologic toxicity is vital to identify high-risk patients and optimize treatment strategies, including radiation techniques and the setting of the treatment fields. Manus *et al*. first reported predictive factors for neutropenia and thrombocytopenia, including concurrent chemotherapy and the amount of irradiated bone marrow [7]. Recently, Terrones *et al*. analyzed a large group of 4055 patients and showed that some dosimetric parameters of radiotherapy, concurrent chemotherapy and primary disease category are related to lymphocytopenia, which is associated with patient survival 1 year after the treatment [2]. However, there have been no large studies that revealed the incidence rates of hematologic toxicities induced by radiation monotherapy and their predictive factors.

This study aimed to reveal the incidence rates of hematologic toxicities induced by radiation monotherapy and develop an easy-to-use method for predicting hematologic toxicities after radiotherapy in a large group of patients.

## MATERIALS AND METHODS

### Patient criteria

This retrospective study included patients aged 18 years or older who received radiation monotherapy at a single institute from 2010 to 2021. Radiation monotherapy was defined as a course of external beam radiotherapy without the use of chemotherapeutic drugs 12 weeks before and after the start of radiotherapy. For patients who received sequential radiotherapies to multiple sites without an interval longer than seven days, the entire sequence of the radiotherapy was defined as a single treatment course. The exclusion criteria were as follows: (i) hematologic malignancies; (ii) the existence of another radiotherapy course in 12 weeks before and after the start of radiotherapy; (iii) lack of hematologic data, including neutrophil count, lymphocyte count, hemoglobin concentration and platelet count, before or after the start of radiotherapy; and (iv) Grade 3 or higher hematologic toxicity in neutrophil count, lymphocyte count, hemoglobin concentration or platelet count at the start of radiotherapy.

### Data collection

We retrospectively reviewed data from 12 weeks before and after the initiation of radiotherapy. We collected data on age, sex, primary disease, radiotherapy details and blood test results.

For radiotherapy, data were collected on the treatment site, overall treatment time (OTT) and radiation field lengths. Radiation field lengths were defined as the longest field length in the X or Y axis of all irradiated fields, which were derived from the radiotherapy record. For patients with multiple irradiation sites on the first day of the treatment course, the sum of the treatment field lengths was recorded as the field length of the patient. We could not evaluate the treatment dose because some patients had multiple prescriptions with different doses and fractionations (i.e. 50 Gy in 25 fractions for the primary site and 30 Gy in 10 fractions for vertebral metastases simultaneously).

To evaluate the hematologic data, we used the neutrophil count, lymphocyte count, hemoglobin concentration and platelet count as hematologic parameters using a hospital information system. Hematologic toxicities were graded as Grade 0–1, Grade 2, Grade 3 or Grade 4 according to the Common Terminology Criteria for Adverse Events (CTCAE) v5.0. To evaluate anemia, only the hemoglobin concentration was considered in determining the toxicity grade because of the lack of transfusion information. The baseline hematologic status was estimated from the most recent blood test data before the start of radiotherapy. The highest grade of hematologic toxicity was estimated from the nadir of each item in the period of 12 weeks after the start of radiotherapy. In addition, the highest grade in each week was documented in the evaluation of changes in hematologic toxicity over time.

### Data analysis

Because of the large number of cases in this study, there was concern that excessive detection of significance in the multivariate analysis could occur, so the cases were divided into training and test groups for analysis. The patients were randomly divided into training and test groups in a 7:3 ratio. The difference between the two groups was evaluated using the Pearson’s chi-square test or Wilcoxon rank-sum test to evaluate the patients’ backgrounds and hematologic toxicity. Continuous variables were divided into four groups based on the quartiles of the training group and nearest round numbers. We divided the treatment sites into four groups based on the data distribution of exploratory analysis in the training group. We defined the very high, high, intermediate and low risk groups as treatment sites where 90% or more, 40–90%, 10–40% and 10% or less of patients showed Grade 3 or higher hematologic toxicities. We analyzed the training group to evaluate the proportion of hematologic toxicities and risk factors for hematologic toxicity, and built a prediction model using an ordinal logistic regression model.

To create a simplified prediction nomogram, we selected parameters with an odds ratio >2.0 or <0.5 and generated nomograms based on the estimated coefficients of an ordinal logistic regression. The nomograms were validated in the training and test groups using receiver operating characteristic (ROC) analysis.

Statistical analyses were performed using Pro 16.2.0 (SAS Institute Inc) and R 4.2.2 (The R Foundation for Statistical Computing Platform).

## RESULTS

### Patient background

A total of 3786 patients met the inclusion criteria and were randomly assigned in a 7:3 ratio to a training group of 2685 patients and a test group of 1101 patients. Patient characteristics are shown in Table 1. The median age of the patients was 68 years in both groups. To simplify the nomogram, the treatment sites were classified into four categories based on the rate of Grade 3 or higher hematologic toxicities (Supplementary Fig. S1): a very high-risk group, including esophageal and whole pelvis irradiation; a high-risk group, including chest (other than esophageal, breast or chest wall irradiation), head and neck, abdomen and pelvis (other than whole pelvis or prostate irradiation) and multiple sites irradiation; an intermediate-risk group, including brain and limb irradiation; and a low-risk group, including breast, chest wall and prostate local irradiation. For radiotherapy, the median OTTs were 40 and 38 days in the training and test groups, respectively. The median radiation field size was 16 cm in both groups. Primary disease sites are shown in Supplementary Table S1. The most common primary disease sites were head and neck cancer (15 and 14% in the training and test groups, respectively), breast cancer (13 and 14%), lung cancer (12 and 14%) and esophageal cancer (11 and 12%). There were no significant differences in the characteristics between the two groups.

**Table 1:** Patient characteristics

**Characteristic**	**Overall *n* = 3786^a^**	**Training *n* = 2685^a^**	**Test *n* = 1101^a^**	***P*-value** ^b^
Age	68 (59, 76)	68 (59, 76)	68 (58, 76)	0.2
Sex				0.7
Female	1684 (44%)	1200 (45%)	484 (44%)	
Male	2102 (56%)	1485 (55%)	617 (56%)	
Treatment site				0.2
Brain	279 (7.4%)	198 (7.4%)	81 (7.4%)	
Head and neck	641 (17%)	468 (17%)	173 (16%)	
Chest (esophagus)	269 (7.1%)	188 (7.0%)	81 (7.4%)	
Chest (breast or chest wall)	463 (12%)	323 (12%)	140 (13%)	
Chest (others)	789 (21%)	541 (20%)	248 (23%)	
Abdomen and pelvis (whole pelvis)	277 (7.3%)	201 (7.5%)	76 (6.9%)	
Abdomen and pelvis (prostate)	250 (6.6%)	183 (6.8%)	67 (6.1%)	
Abdomen and pelvis (others)	490 (13%)	355 (13%)	135 (12%)	
Limb	60 (1.6%)	34 (1.3%)	26 (2.4%)	
Multiple sites, others	268 (7.1%)	194 (7.2%)	74 (6.7%)	
Risk group of treatment site				0.8
Low	713 (19%)	506 (19%)	207 (19%)	
Intermediate	339 (9.0%)	232 (8.6%)	107 (9.7%)	
High	2188 (58%)	1558 (58%)	630 (57%)	
Very high	546 (14%)	389 (14%)	157 (14%)	
Baseline neutrophil count (10^3^/μl)	3.95 (2.89, 5.54)	3.97 (2.91, 5.53)	3.89 (2.81, 5.57)	0.6
Baseline lymphocyte count (10^3^/μl)	1.47 (1.10, 1.91)	1.48 (1.09, 1.90)	1.45 (1.11, 1.91)	0.8
Baseline hemoglobin concentration (g/dl)	12.4 (11.1, 13.5)	12.4 (11.1, 13.5)	12.4 (11.1, 13.5)	0.8
Baseline platelet count (10^3^/μl)	228 (185, 285)	228 (185, 284)	230 (185, 285)	0.6
History of radiotherapy	328 (8.7%)	225 (8.4%)	103 (9.4%)	0.3
OTT (days)	40 (17, 47)	40 (17, 47)	38 (16, 47)	0.11
Size of radiation field (cm)	16 (10, 21)	16 (10, 21)	16 (10, 21)	0.8

^a^Median (IQR), *n* (%);

^b^Wilcoxon rank sum test, Pearson’s Chi-squared test

### Hematologic toxicity

**Fig. 1:**
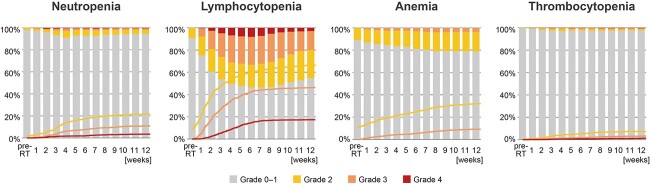
The rates of hematologic toxicities. Bar graphs show the rates of the highest grade of hematologic toxicity observed in each week from the start of radiotherapy. Line graphs show Kaplan–Meier plots of cumulative incidences of hematologic toxicities.

Table 2 shows the details of hematologic toxicities in the training and test groups. In these groups, Grade 3 or higher hematological toxicities were observed in 9.5 and 10.1% of patients with neutropenia, 44.7 and 44.5% with lymphocytopenia, 8.5 and 8.1% with anemia and 3.4 and 2.5% with thrombocytopenia, respectively. There were no significant differences in the characteristics between the two groups. Figure 1 shows the changes and cumulative incidence of hematologic toxicities in all patients during the 12 weeks’ period after the start of radiotherapy. The most severe neutropenia and lymphocytopenia were observed in 8 weeks from the start of radiotherapy.

**Table 2:** Details of hematologic toxicities in each patient group

**Characteristic**	**Overall *n* = 3786**	**Training *n* = 2685**	**Test *n* = 1101**	***P*-value** ^a^
Neutrophil				0.7
Grade 0–1	3036 (80%)	2155 (80%)	881 (80%)	
Grade 2	384 (10%)	275 (10%)	109 (9.9%)	
Grade 3	247 (6.5%)	176 (6.6%)	71 (6.4%)	
Grade 4	119 (3.1%)	79 (2.9%)	40 (3.6%)	
Lymphocyte				>0.9
Grade 0–1	1380 (36%)	975 (36%)	405 (37%)	
Grade 2	717 (19%)	511 (19%)	206 (19%)	
Grade 3	1076 (28%)	767 (29%)	309 (28%)	
Grade 4	613 (16%)	432 (16%)	181 (16%)	
Hemoglobin				0.8
Grade 0–1	2664 (70%)	1881 (70%)	783 (71%)	
Grade 2	806 (21%)	577 (21%)	229 (21%)	
Grade 3	316 (8.3%)	227 (8.5%)	89 (8.1%)	
Platelet				0.2
Grade 0–1	3522 (93%)	2490 (93%)	1032 (94%)	
Grade 2	145 (3.8%)	103 (3.8%)	42 (3.8%)	
Grade 3	73 (1.9%)	60 (2.2%)	13 (1.2%)	
Grade 4	46 (1.2%)	32 (1.2%)	14 (1.3%)	

^a^Pearson’s Chi-squared test

### Predictive nomogram for hematologic toxicity

**Fig. 2:**
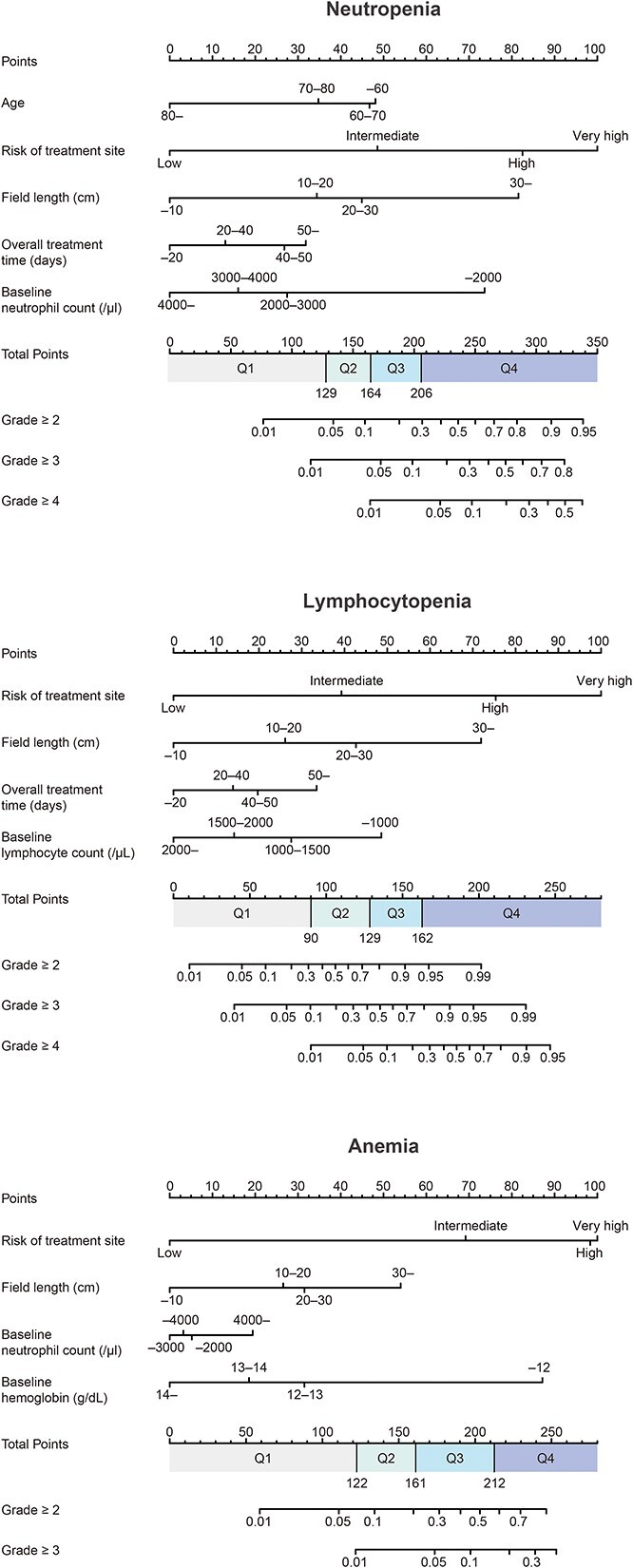
Nomograms for prediction of hematologic toxicity. Q1, Q2, Q3 and Q4 show the subgroups divided according to the quartiles of the total nomogram scores in the training cohort.

**Fig. 3:**
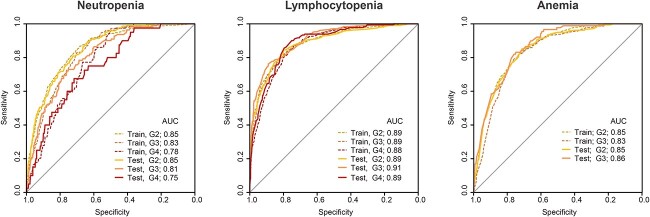
Receiver operating characteristic (ROC) plots of the nomograms for predicting hematologic toxicities. *AUC* = area under the ROC curve. G2–G4 represent the toxicity grades according to the CTCAE version 4.0.

**Fig. 4:**
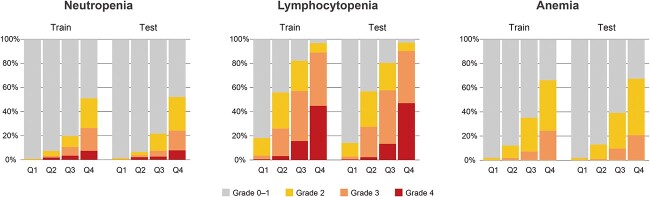
Rates of hematologic toxicities in patient subgroups. Q1, Q2, Q3 and Q4 show the subgroups divided according to the quartiles of the total nomogram scores in the training cohort.

Table 3 shows the results of ordinal logistic regression analysis. We identified six factors for neutropenia grade, nine for lymphocytopenia grade and six for anemia grade with statistical significance. For thrombocytopenia, the statistical model did not converge because of a small number of events. Among the factors with *P*-values smaller than 0.05, we selected factors with odds ratios >2.0 or <0.5 to construct nomograms (Fig. 2). The following risk factors were identified: neutropenia; age, the risk of the treatment site, radiation field length, OTT and baseline neutrophil count. Lymphocytopenia; treatment site risk, radiation field length, OTT and baseline lymphocyte count. Anemia; treatment site risk, radiation field length, baseline neutrophil count and baseline hemoglobin concentration. Figure 3 shows the ROC curves of the nomograms for hematologic toxicities. For predicting Grade 2–4 hematologic toxicities, the area under the ROC curve (AUC) values of the nomograms in the training and the test groups were 0.78–0.85 and 0.75–0.85 for neutropenia, 0.88–0.89 and 0.89–0.91 for lymphocytopenia and 0.83–0.85 and 0.85–0.86 for anemia, respectively. For practical predictive use, we divided each patient group into four risk categories (Q1, Q2, Q3 and Q4) based on the total score quartile of the nomograms (Fig. 2). Figure 4 shows the hematologic toxicity rates in each subgroup based on the nomograms.

**Table 3:** The result of ordinal logistic regression analysis in the training group

		**Neutropenia**		**Lymphocytopenia**		**Anemia**	
**Characteristic**	***N***	**OR**	***P*-value**	**OR**	***P*-value**	**OR**	***P*-value**
Age (years)							
–60	697	—		—		—	
60–70	768	0.98 (0.74–1.29)	0.9	0.96 (0.77–1.20)	0.7	1.04 (0.79–1.37)	0.8
70–80	805	0.64 (0.48–0.86)	**0.003**	0.85 (0.68–1.06)	0.2	1.02 (0.78–1.34)	0.9
80–	415	0.22 (0.14–0.34)	**<0.001**	0.53 (0.41–0.69)	**<0.001**	0.76 (0.56–1.04)	0.091
Sex							
Female	1200	—		—		—	
Male	1485	1.02 (0.79–1.30)	>0.9	1.27 (1.06–1.52)	**0.01**	1.1 (0.89–1.36)	0.4
Risk group of treatment site							
Low	506	—		—		—	
Intermediate	232	4.42 (2.24–8.83)	**<0.001**	5.83 (3.99–8.54)	**<0.001**	8.75 (4.77–16.7)	**<0.001**
High	1558	11.6 (7.06–20.3)	**<0.001**	37.1 (27.6–50.3)	**<0.001**	20.1 (12.1–35.3)	**<0.001**
Very high	389	19.1 (11.5–33.6)	**<0.001**	133 (93.4–190)	**<0.001**	26.4 (15.5–47.1)	**<0.001**
Baseline neutrophil count (/μl)							
≥ 4000	1326	—		—		—	
4000–3000	635	1.59 (1.19–2.11)	**0.001**	0.95 (0.78–1.16)	0.6	0.62 (0.49–0.80)	**<0.001**
3000–2000	545	2.19 (1.64–2.92)	**<0.001**	1.01 (0.81–1.25)	>0.9	0.57 (0.43–0.74)	**<0.001**
< 2000	179	7.46 (5.12–10.9)	**<0.001**	1.41 (1.01–1.97)	**0.046**	0.61 (0.40–0.90)	**0.015**
Baseline lymphocyte count (/μl)							
≥ 2000	550	—		—		—	
1500–2000	768	0.92 (0.67–1.27)	0.6	1.95 (1.53–2.49)	**<0.001**	0.98 (0.72–1.32)	0.9
1000–1500	852	1.28 (0.93–1.76)	0.13	3.78 (2.97–4.82)	**<0.001**	1.12 (0.84–1.51)	0.4
< 1000	515	1.51 (1.04–2.18)	**0.03**	9.94 (7.49–13.2)	**<0.001**	1.59 (1.15–2.19)	**0.005**
Baseline hemoglobin concentration (g/dl)							
≥ 14	480	—		—		—	
13–14	518	0.97 (0.67–1.41)	0.9	1.17 (0.90–1.54)	0.2	1.91 (1.24–3.00)	**0.004**
12–13	585	1.42 (0.98–2.06)	0.062	1.09 (0.84–1.42)	0.5	2.96 (1.96–4.57)	**<0.001**
< 12	1102	1.23 (0.87–1.74)	0.2	1.18 (0.92–1.51)	0.2	17 (11.7–25.5)	**<0.001**
Baseline platelet count (10^3^/μl)							
≥ 250	1031	—		—		—	
200–250	741	0.97 (0.72–1.29)	0.8	0.79 (0.65–0.97)	**0.028**	0.88 (0.68–1.13)	0.3
150–200	676	1.11 (0.82–1.49)	0.5	0.83 (0.67–1.02)	0.078	0.86 (0.66–1.12)	0.3
< 150	237	1.19 (0.79–1.77)	0.4	1.06 (0.78–1.43)	0.7	1.24 (0.86–1.77)	0.2
Past radiotherapy							
No	2460	—		—		—	
Yes	225	0.71 (0.42–1.15)	0.2	1.64 (1.24–2.18)	**<0.001**	0.91 (0.64–1.28)	0.6
OTT (days)							
1–20	762	—		—		—	
21–40	542	1.5 (1.02–2.20)	**0.037**	2.04 (1.61–2.61)	**<0.001**	0.6 (0.44–0.82)	**0.001**
41–50	862	2.32 (1.67–3.26)	**<0.001**	2.68 (2.14–3.37)	**<0.001**	0.75 (0.57–0.99)	**0.042**
51–	519	2.78 (1.93–4.00)	**<0.001**	5.16 (3.95–6.77)	**<0.001**	1.68 (1.24–2.27)	**<0.001**
Size of radiation field (cm)							
≤ 10	718	—		—		—	
10–20	1087	2.53 (1.77–3.67)	**<0.001**	3.57 (2.88–4.43)	**<0.001**	2.33 (1.77–3.07)	**<0.001**
20–30	590	3.37 (2.28–5.05)	**<0.001**	7.91 (6.09–10.3)	**<0.001**	2.49 (1.81–3.44)	**<0.001**
> 30	290	10.1 (6.66–15.4)	**<0.001**	31.4 (22.6–43.8)	**<0.001**	4.93 (3.43–7.10)	**<0.001**

OR = odds ratio. OR is shown with a 95% confidence interval. For thrombocytopenia, the statistical model did not converge because of a small number of events.

## DISCUSSION

In this study, we analyzed data from 2685 patients in the training group of the 3786 patients treated with radiation monotherapy to evaluate treatment-related hematologic toxicity and its predictive factors. We created predictive nomograms for neutropenia, leukocytopenia and anemia based on an ordinal logistic analysis. These nomograms were evaluated in 1101 patients in the independent test group and showed high predictive ability in the ROC analysis. This is one of the largest studies to evaluate radiation-induced hematologic toxicities, and the first report to propose predictive nomograms in patients treated with radiation monotherapy.

In cancer treatment, hematologic toxicity is often observed for various reasons, including bleeding, chronic inflammation, tumor invasion into the bone marrow, radiation therapy and chemotherapy. Hematologic toxicity during cancer treatment can compromise patient feasibility and is associated with poor outcomes [2]. Revealing predictive factors is helpful in minimizing hematologic toxicities when planning radiation therapy; however, few publications have described the probability of hematologic toxicity and predictive factors in the entire cancer population. Moreover, most of the existing reports included patients who received sequential or concurrent chemotherapy, which makes it difficult to evaluate the effect of radiotherapy on hematologic toxicities because the use of chemotherapy is an obvious risk factor for hematologic toxicities [2]. In this study, we provide basic data on the incidence of hematologic toxicities after radiotherapy without chemotherapy. Moreover, we analyzed radiation records and identified the predictive factors for neutropenia, lymphocytopenia and anemia. Each predictive factor is discussed below.

First, we found that the treatment site was associated with hematologic toxicities. We divided the treatment sites into four risk groups based on an exploratory analysis in the training cohort first to make the nomograms easy to use. For example, we defined breast, chest wall and prostate local irradiations as low risk and esophageal and whole pelvic irradiation as very high risk. This classification was well consisted with the distribution of proliferating bone marrow tissue described in the existing report using 18F-fluoro-L-deoxythymidine scanning [8].

Second, the length of the radiation field was found to be associated with hematologic toxicities. In existing reports, Manus *et al*. showed that the irradiated proportion of vertebral bone was associated with a higher grade of hematologic toxicity [7]. Recent studies have shown some dose-volume histogram (DVH) parameters of the vertebral and pelvic bones [9–14]. In lymphocytopenia, the total irradiated volume of the body and body structures other than hematopoietic tissues is also related to lymphocyte cell death because lymphocytes have very high radiosensitivity, and interphase death occurs when passing through the irradiated area [6, 15]. The radiation field length is easy to measure and can be used as a surrogate marker for evaluating the detailed DVH parameters.

Among other factors, the OTT was a predictive factor for neutropenia and lymphocytopenia. This may be related to the higher radiation dose to hematopoietic tissues and the higher probability of lymphocytes passing through the radiation field [16]. The baseline cell count was also associated with hematologic toxicity. Considering these factors, it may be better to choose radiotherapy over a short course with fewer fractions to avoid severe hematologic toxicity. There is a report in postoperative breast cancer patients that hypo-fraction radiotherapy with 15 or 16 fractions, which is considered to have the same treatment effect as conventional 25 fractions, reduced lymphocytopenia after radiotherapy [5]. In choosing the optimal dose and fractionation, it may be possible to reduce hematologic toxicities without compromising treatment efficacy. It may also be an option to shrink the radiation field by optimizing target delineation using advanced imaging modalities and shrinking margins using image-guided radiotherapy.

In evaluating the nomograms, we found high AUC values (neutropenia; 0.75–0.85, lymphocytopenia; 0.89–0.91 and anemia; 0.85–0.86, respectively). By categorizing the variables and excluding factors with a lower contribution to prediction, we could make the nomograms simple, easy to use and robust for validation in a test patient dataset. The AUC values were almost the same as those of the models using all factors, without exclusion or quantization (data not shown). In future studies, it will be necessary to evaluate our nomograms using independent patient cohorts from multiple institutes. In addition, it is necessary to consider modifying supporting interventions for high-risk patients. For example, the incidence rates of Grade 3 or higher neutropenia in the test cohort were 0.4, 3.6, 7.5 and 24.2% in subgroups Q1, Q2, Q3 and Q4, respectively. It might be a treatment option to use agents, such as granulocyte colony-stimulating factor, in the early stages to prevent severe neutropenia.

This study had several limitations. First, we were unable to determine radiation doses for each patient. This is because some patients received multiple prescriptions of different doses and fractionations during the course of treatment. Second, this study did not include DVH parameters of the bone marrow, other organs or the whole body because it was a database study. The information could be obtained by setting up a subgroup study considering the treatment contents and available information, but this was not possible for the entire population of this study. Third, patients treated with chemotherapy were excluded only if there was a record of chemotherapy 12 weeks before and after the start of radiotherapy because of data availability. Therefore, we cannot exclude the possibility that a past history of chemotherapy may have influenced hematologic toxicity.

In conclusion, we established predictive nomograms for neutropenia, leukocytopenia and anemia and demonstrated high reproducibility when validated in an independent cohort of patients.

## Supplementary Material

Supplementary_materials_rrad069

## Data Availability

The datasets used in the current study are available from the corresponding author upon reasonable request, with permission from the institution.

## PRESENTATION AT A CONFERENCE

This study’s abstract will be presented at the 35th Annual Meeting of the Japanese Society for Radiation Oncology, 2023.
