# Supplementary material for: Acute hematologic toxicity of radiation therapy – a comprehensive analysis and predictive nomogram
Source: J Radiat Res. 2023 Sep 22;64(6):954–61. doi: 10.1093/jrr/rrad069 (PMC10665302; doi:10.1093/jrr/rrad069)
Supplement: Supplementary_materials_rrad069 [file supplementary_materials_rrad069.zip › Supplementary_materials_rrad069/20230809_Table_Sup1.docx]

Supplementary Table 1. Additional patient characteristics.

| **Characteristic** | **Overall**  n = 3,786^1^ | **training**  n = 2,685^1^ | **test**  n = 1,101^1^ | **p-value**^2^ | |
| --- | --- | --- | --- | --- | --- |
| Primary disease site |  |  |  | 0.7 | |
| Brain | 172 (4.5%) | 130 (4.8%) | 42 (3.8%) |  | |
| Head and neck | 570 (15%) | 414 (15%) | 156 (14%) |  | |
| Lung | 483 (13%) | 332 (12%) | 151 (14%) |  | |
| Breast | 502 (13%) | 350 (13%) | 152 (14%) |  | |
| Esophagus | 436 (12%) | 306 (11%) | 130 (12%) |  | |
| Other intestinal tracts | 47 (1.2%) | 34 (1.3%) | 13 (1.2%) |  | |
| Liver, biliary tract, and pancreas | 98 (2.6%) | 71 (2.6%) | 27 (2.5%) |  | |
| Uterus | 350 (9.2%) | 249 (9.3%) | 101 (9.2%) |  | |
| Prostate | 324 (8.6%) | 240 (8.9%) | 84 (7.6%) |  | |
| Urinary tract | 146 (3.9%) | 108 (4.0%) | 38 (3.5%) |  | |
| Bone and soft tissue | 83 (2.2%) | 58 (2.2%) | 25 (2.3%) |  | |
| Skin | 46 (1.2%) | 35 (1.3%) | 11 (1.0%) |  | |
| Benign diseases | 115 (3.0%) | 77 (2.9%) | 38 (3.5%) |  | |
| Others | 113 (3.0%) | 74 (2.8%) | 39 (3.5%) |  | |
| Unknown | 301 (8.0%) | 207 (7.7%) | 94 (8.5%) |  | |
| ^1^Median (IQR); n (%) |  |  |  |  | |
| ^2^Wilcoxon rank sum test; Pearson's Chi-squared test | | | |  | |
|  |  |  |  |  | |
|  |  |  |  |  | |
|  | | | | |  |
|  | | | | |  |
