# Supplementary figures and images for: Acute hematologic toxicity of radiation therapy – a comprehensive analysis and predictive nomogram
Source: J Radiat Res. 2023 Sep 22;64(6):954–61. doi: 10.1093/jrr/rrad069 (PMC10665302; doi:10.1093/jrr/rrad069)

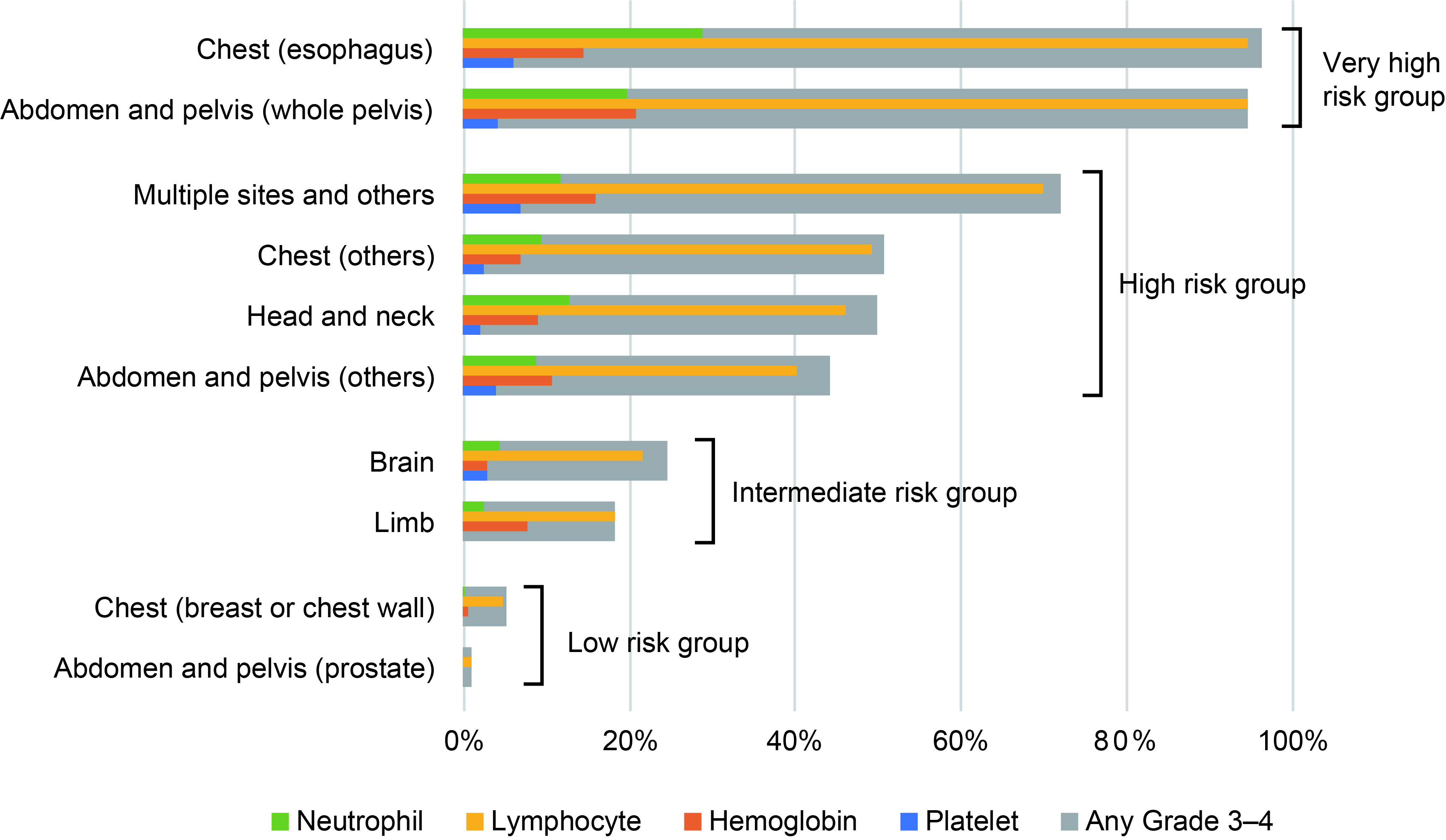

Supplement: Supplementary_materials_rrad069 [file supplementary_materials_rrad069.zip › Supplementary_materials_rrad069/20230809_FigureSup1.tif]
